# Hit-Gel: Streamlining in-gel protein digestion for high-throughput proteomics experiments

**DOI:** 10.1038/s41598-018-26639-3

**Published:** 2018-06-05

**Authors:** Corné Swart, Silvia Martínez-Jaime, Michal Gorka, Kerstin Zander, Alexander Graf

**Affiliations:** Max Planck Institute of Molecular Plant Physiology, 14476 Potsdam-Golm, Germany

## Abstract

In-gel digestion has been used as a standard method for the preparation of protein samples for mass spectrometry analysis for over 25 years. Traditional in gel-digestion procedures require extensive sample handling, are prone to contamination and not compatible with high-throughput sample preparation. To address these shortcomings, we have modified the conventional in-gel digestion procedure for high-throughput proteomics studies. The modified method, termed “High Throughput in Gel digestion” (HiT-Gel), is based on a 96-well plate format which results in a drastic reduction in labour intensity and sample handling. Direct comparison revealed that HiT-Gel reduces technical variation and significantly decreases sample contamination over the conventional in-gel digestion method. HiT-Gel also produced superior results when a single protein band was excised from a gel and processed by in-gel digestion. Moreover, we applied Hit-Gel for a mass spectrometry analysis of *Arabidopsis thaliana* protein complexes separated by native PAGE in 24 fractions and four biological replicates. We show that the high throughput capacity of HiT-Gel facilitates large scale studies with high sample replication or detailed fractionation. Our method can easily be implemented as it does not require specialised laboratory equipment.

## Introduction

In-gel digestion is a standard method to prepare proteins obtained from biological samples for mass-spectrometry (MS)-based analysis. It is commonly used to facilitate the identification of proteins from specific bands or spots following 1D or 2D PAGE analysis. Moreover, the fractionation of complex protein extracts by 1D SDS page followed by in-gel digestion is a standard approach for reducing the complexity of peptide mixtures and increasing proteome coverage in “shotgun” measurements^1–4^.

With the development of faster and more sensitive mass spectrometers, the complexity of protein extracts became less of a concern. With the technical ability to quantify several thousand proteins in a single sample, sample preparation methods like eFASP^5,6^, “single shot” ^7^, In-StageTip^8^ and GASP^9^ gained popularity. These methods are easy to use, minimize sample handling and can even be automated, making them indispensable to high-throughput proteomics experiments. As a consequence the traditional fractionation-based approaches using 1D and 2D gel electrophoresis are now becoming less favored^4^. Although these new methods offer multiple benefits, they are not amenable to all experimental requirements and as a result the traditional methods remain in use. The classic 1D gel electrophoresis and subsequent in-gel digestion procedure, introduced by Rosenfeld *et al*.^2^ is an excellent example of such a traditional method. With its modification for improved compatibility with MS^1^, this robust method provides an opportunity to visually inspect the quality of protein samples when combined with a staining approach. Additionally the method removes chemical compounds that could interfere with the digestion and downstream mass spectrometric measurements^1^. In-gel digestion also remains essential in workflows to identify and quantify by mass spectrometry the components of protein complexes fractionated by native PAGE. A major disadvantage of the traditional in-gel digestion protocols is that they demand extensive sample handling, are prone to contamination (especially by keratin) and cannot easily be scaled up for high throughput sample preparation^1,10^.

In this article a modified protocol for in-gel protein digestion is presented. Considerable modifications to the original sample preparation workflow (henceforth “conventional method”)^1,2^ has led to a drastic decrease in labour intensity and sample handling, in turn offering scalability of 1D gel based proteomics for high throughput samples preparation and thereby enabling large-scale gel-based proteomics studies. The modified method was named “High Throughput in Gel digestion” (HiT-Gel). A key change introduced to HiT-Gel is that fractions are processed as intact gel pieces rather than being diced into small cubes of roughly 1 mm × 1 mm edge length as in the conventional method^1,2^. It was suggested that this step was critical for protein digestion by facilitating the diffusion of the protease into the gel pieces^11^. Data presented here suggests that cutting the gel fractions into smaller cubes does in fact not have any positive influence on the recovery of peptides from the gel and the number of identified proteins. The data also reveals that the additional cutting step significantly increases the risk of sample contamination. As gel fractions are kept intact in the HiT-Gel method, the risk of picking up and discarding small gel pieces with a pipette tip during the repeated exchange of solutions is reduced in comparison to the conventional method. Another important alteration to the conventional method is that the gel pieces are no longer transferred to microfuge tubes but to 96-well plates, where all sample preparation steps are performed with the aid of multichannel pipettes. Three datasets including two direct comparisons of the conventional in-gel digestion method and the HiT-Gel method are presented. The results show that HiT-Gel is a major advancement with regards to reduction of workload and sample contamination and increased reproducibility. It facilitates high-throughput sample preparation, which could especially be of interest for quantitative, gel-based studies of native protein complexes with detailed fractionation and sample replication.

## Results and Discussion

### Direct comparison between the HiT-Gel and the conventional in-gel digestion method

To allow a direct comparison of the conventional in-gel digestion method and the HiT-Gel method, samples were prepared simultaneously for both methods using the three technical replicates for each method. Proteins were extracted from 3 week old *Arabidopsis thaliana* rosettes. Six replicates of 100 µg total protein were separated by 1D SDS PAGE on the same gel and proteins were visualized by coomassie staining. All six gel lanes were sliced into 8 fractions of nearly equivalent size (Supplementary Figure S1). At this stage samples from three non-adjacent lanes were subjected to the conventional method^1,2^, wherein each of the gel pieces was further subdivided into cubes of approximately 1 mm side length. These cubes were subjected to in-gel protein digestion and peptide elution in individual microfuge tubes as previously described. The remaining samples were not subdivided into smaller cubes. Instead the gel fractions were transferred to 96-well plates. Solution volumes for the in-gel digestion procedure were adjusted for the HiT-Gel method to ensure complete submersion of the gel pieces (Supplementary Methods). A standard multi-channel pipette was utilised to facilitate the exchange of solutions, leading to reduced sample handling and faster processing times.

**Figure 1:**
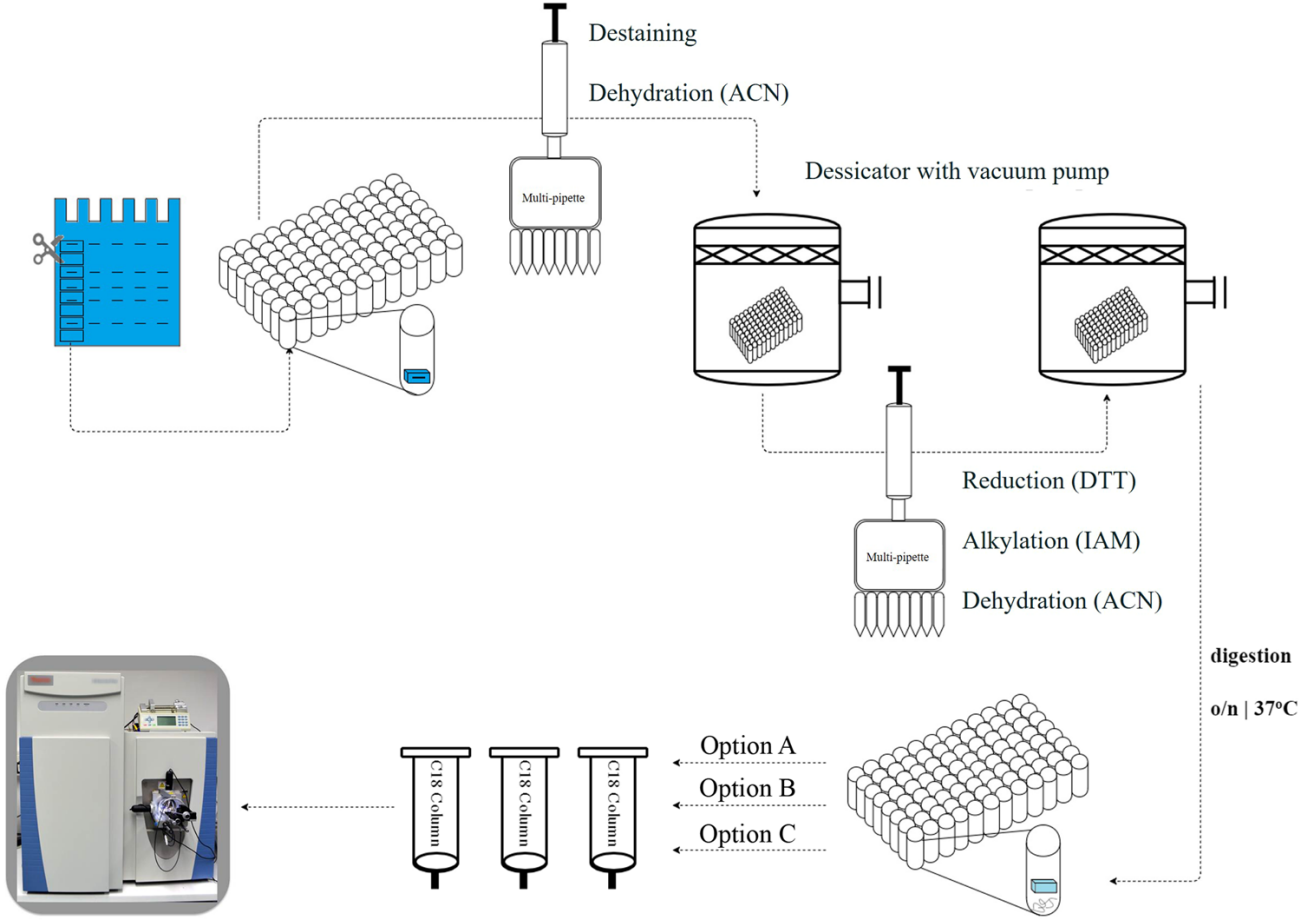
Schematic workflow of the HiT-Gel method. Protein extracts are separated by means of PAGE before fractionation of the gel. Intact gel slices are transferred to 96-well plates, where all subsequent liquid exchange steps are performed using a multichannel pipette. After the gel pieces have been destained and dehydrated, residual liquid is evaporated with the aid of a desiccator vacuum pump. The samples are then reduced, alkylated and the gel pieces dehydrated as per the conventional protocol. Trypsin is added for overnight digestion at 37 °C. At this stage the protocol can be adjusted based on the availability of equipment. Option A: Digested peptides are transferred to a new 96-well plate and dried down in a vacuum centrifuge with a swing out rotor. Option B: Digested peptides are transferred to 2 ml reaction tubes using a multichannel pipette with adjustable spacers, and dried down in a vacuum centrifuge. Option C: Digested peptides are transferred to 2 ml reaction tubes using a standard pipette and dried down using a vacuum centrifuge. The peptides are then desalted and analysed by LC-MS/MS. For more details, please refer to the Supplementary Methods.

The workflow of the HiT-GEL method (Fig. 1) can be further optimized depending on the available laboratory equipment. Following overnight incubation of the gel pieces with trypsin, removal of acetonitrile from the peptide containing solution prior to desalting of the peptides can be performed according to one of three options (Supplementary Methods). The first and most efficient option (A) requires a vacuum centrifuge equipped with a 96-well plate compatible swing-out rotor. The peptide containing solution is transferred from the plate containing the gel pieces to a second plate using a multi-channel pipette. After the peptide elution steps, this second plate is placed into the vacuum centrifuge to remove acetonitrile from the solution. The second option (B) employs a multi-channel pipette with adjustable spacers. Using this pipette, the peptide containing solution can be transferred from the 96-well plate to racks carrying single 2 ml reaction tubes with little effort. Vacuum centrifugation is then performed in the single tubes using a standard rotor. In the most labour-intensive option (C), transferring the eluted peptides from each well of the plate to single reaction tubes, is performed using a standard pipette. The peptides collected from both in-gel digestion approaches were desalted and subjected to LC-MS/MS analysis. Samples were processed in the same order as on the gel. Hence, samples from both methods were run alternating to avoid position effects. To ensure that no difference in mass spectrometer performance occurred, we analysed the mass error (Supplementary Figure S2) and created an overview of the MS (peak count), MS/MS (count) and the signal intensity for all of the measurements (Supplementary Dataset 1).

**Figure 2:**
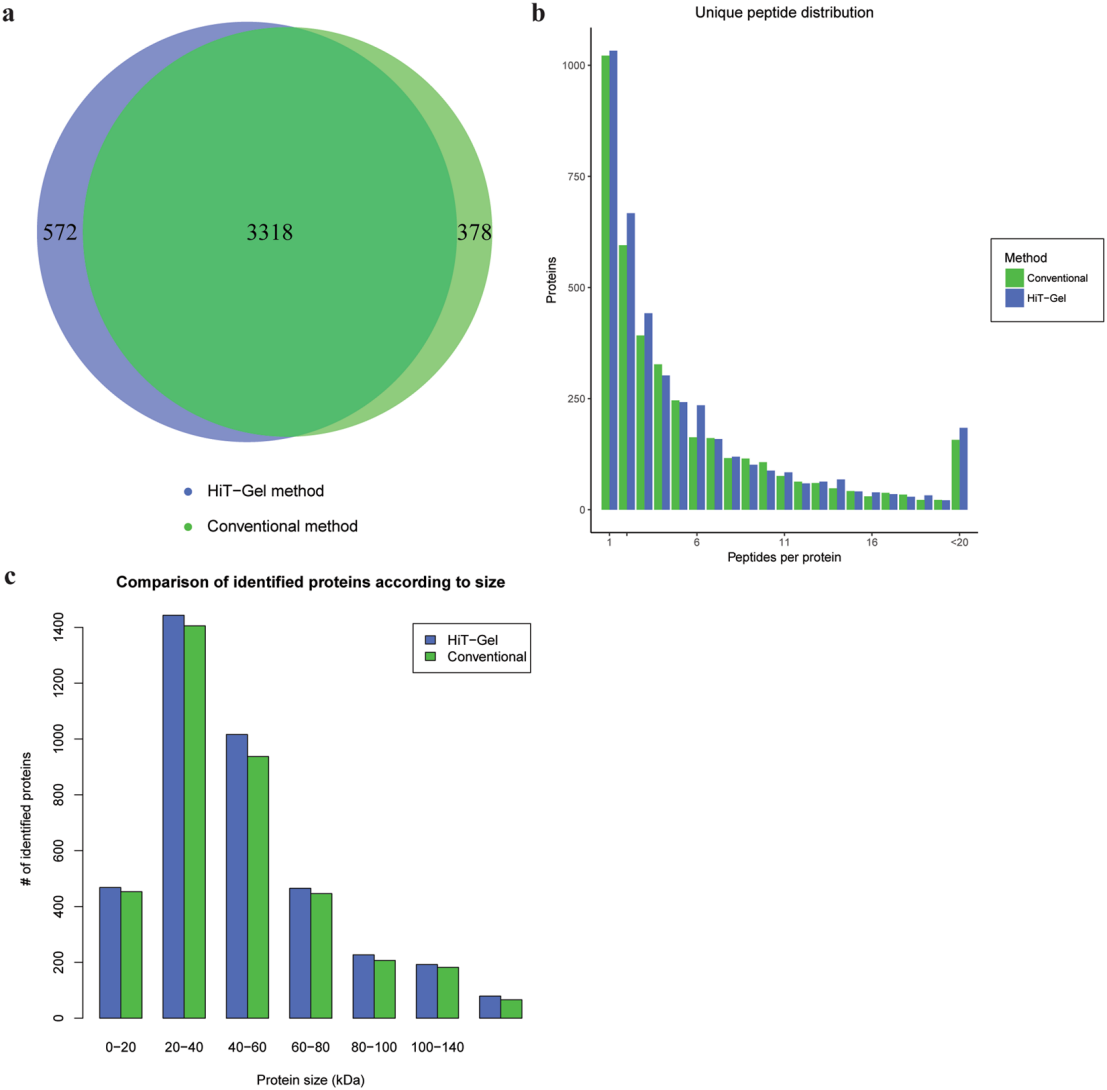
Benchmarking the performance of the HiT-Gel method against the conventional method. (**a**) The overlap between the proteins quantified in each of the methods is presented as a Venn diagram. **(b)** The ability of each method to measure unique peptides per protein on average was calculated. To visually represent the data, a bar plot was produced that shows the difference between the average number of unique peptides per protein for the HiT-Gel method and the conventional method. **(c)** The ability of each method to quantify proteins across the size range of 0 to 600 kDa is represented as a barplot.

Following analysis of the spectral data, the total number of quantifiable peptides obtained from the mascot search engine with the HiT-Gel method (49501), was approximately 5% higher than with the conventional method (47160) (Table 1). A similar increase was observed on the protein level, where 3890 proteins were detected and quantified using the Hit-Gel method and 3696 using the conventional method (Supplementary Dataset 2). The overlap between the quantified proteins of the two methods was 77.7% (Fig. 2a). The average detection of peptides per protein was slightly higher in the Hit-Gel method (5.4) compared to the conventional method (5.3). This is also reflected in the difference of protein numbers with specific peptide count between the two methods (Fig. 2b). The slightly higher number of identified peptides and proteins can be attributed to an approximately 10% higher ion intensity in the MS runs of samples prepared using the HiT-Gel method when compared to the conventional method (Table 1 and Fig. 2c). Considering that samples for both methods contained the same starting material, the increased ion intensity indicates a better recovery of peptides from the gel matrix when the HiT-Gel method was employed. These findings strongly suggest that the subdivision of gel fractions into small cubes as performed in the conventional method is not required for efficient protein digestion and peptide elution^1,2^. It has been suggested that the peptidases used for in-gel digestion enter the gel pieces by diffusion^11–13^. It is conceivable that adequate diffusion of peptidases into the gel can take place during an overnight incubation and that the surface area of the gel pieces does not limit the protein digestion. In fact, the increase in surface area introduced by cutting the gel fractions into small cubes could account for the lower signal intensity and number of identified proteins observed in the conventional method. It is possible that on the surface and especially at the edges created by cutting the gel using razor blades or scalpels proteins diffuse from the gel matrix into the solution during the multiple solution exchanges preceding the digest, thereby leading to a loss of sample material and lower peptide recovery from the gel.

**Table 1:** Comparison of the HiT-Gel and conventional method on a complex protein sample.

	HiT-Gel	Conventional
Number of quantified proteins	3890	3696
Intensity of quantified proteins	6,7e11	6,2e11
Total number of contaminants	76	75
Number of quantified contaminants	37 (31 = keratin)	33 (27 = keratin)
Intensity of quantified contaminants	1.19e10	4.17e10
Intensity of quantified keratins	1,2e9	1,1e10
Peptides used for quantification	49501	47160

The two datasets obtained from the plant extracts separated by SDS-PAGE were compared in terms of quantified peptides, proteins and contaminants. Signal intensities are also reported for the quantified proteins and contaminants. Protein identification was performed in Mascot (No protein grouping, unique peptides only, 1% FDR, Mascot ion score >25) before being reimported into Progenesis QI.

Quantitative proteomic studies require sample preparation methods that are highly reproducible and introduce as little technical variation to a dataset as possible. A covariance analysis was performed to determine the technical variation in the data obtained from both in-gel digestion methods. The Hit-Gel samples had a slightly lower sample-to-sample variation as reflected by a shift in the coefficient of variation density plot (Supplementary Figure S3). A lower technical variation was expected in samples prepared with HiT-Gel method. The bivariate correlation was also investigated among the replicates of the conventional method (Supplementary Table S1) and the HiT-Gel method (Supplementary Table S2). Furthermore, we also compared the technical replicates between the methods using the same Pearson correlation coefficient method (Supplementary Table S3). The data showed that both of the methods are very comparable on the level of the individual replicates. In addition to the low technical variance, the HiT-Gel method employs a multi-channel pipette that enables fast and parallel exchange of solutions, and consequently markedly reduces processing time and sample handling.

Sample contamination, especially with keratin, has been highlighted as a downside of the conventional in-gel digestion method^1^. The modifications introduced to establish the HiT-Gel method leads to a dramatic decrease in the sample contamination. While the number of detected contaminants is comparable in both methods (Table 1), the total ion intensity of all contaminants is 71.5% lower in the HiT-Gel method than in the conventional method (Table 1 & Supplementary Figure S4). The observed decrease in ion intensity of the contaminants was also found to be significant (T-test, pval <0.05). This effect is even more pronounced when keratin is considered separately. In this case a reduction in ion intensity by 82.7% in the Hit Gel compared to the conventional method was observed.

Besides full-proteome analysis, in-gel digestion has been used widely to analyse specific protein bands or spots from gels, for example to identify interaction partners following Co-Immunoprecipitation (CoIP) experiments. To test whether the HiT-Gel method can also be used for such tasks, 50 ng of BSA were subjected to SDS PAGE. As before, six technical replicates were created. Following coomassie staining, the bands corresponding to BSA were cut from the gel and three replicates were used for the conventional in-gel digestion or the HiT-Gel method, respectively. The resulting peptide mixtures were subjected to HPLC-MS/MS analysis and data was analysed as described above. Using the HiT-Gel method, total ion intensities observed for BSA were approximately 1.8 fold higher compared to the conventional method (Supplementary Table S4). The higher ion intensities also translate into better protein coverage: 33 BSA peptides were quantified using the conventional method while 38 were quantified using HiT-Gel (Supplementary Table S4). Taken together, these results confirm that HiT-Gel allows a better peptide recovery from the gel matrix not only in complex samples but also when single protein spots or bands are isolated from a gel.

### High throughput sample preparation for native protein complex analysis

**Figure 3:**
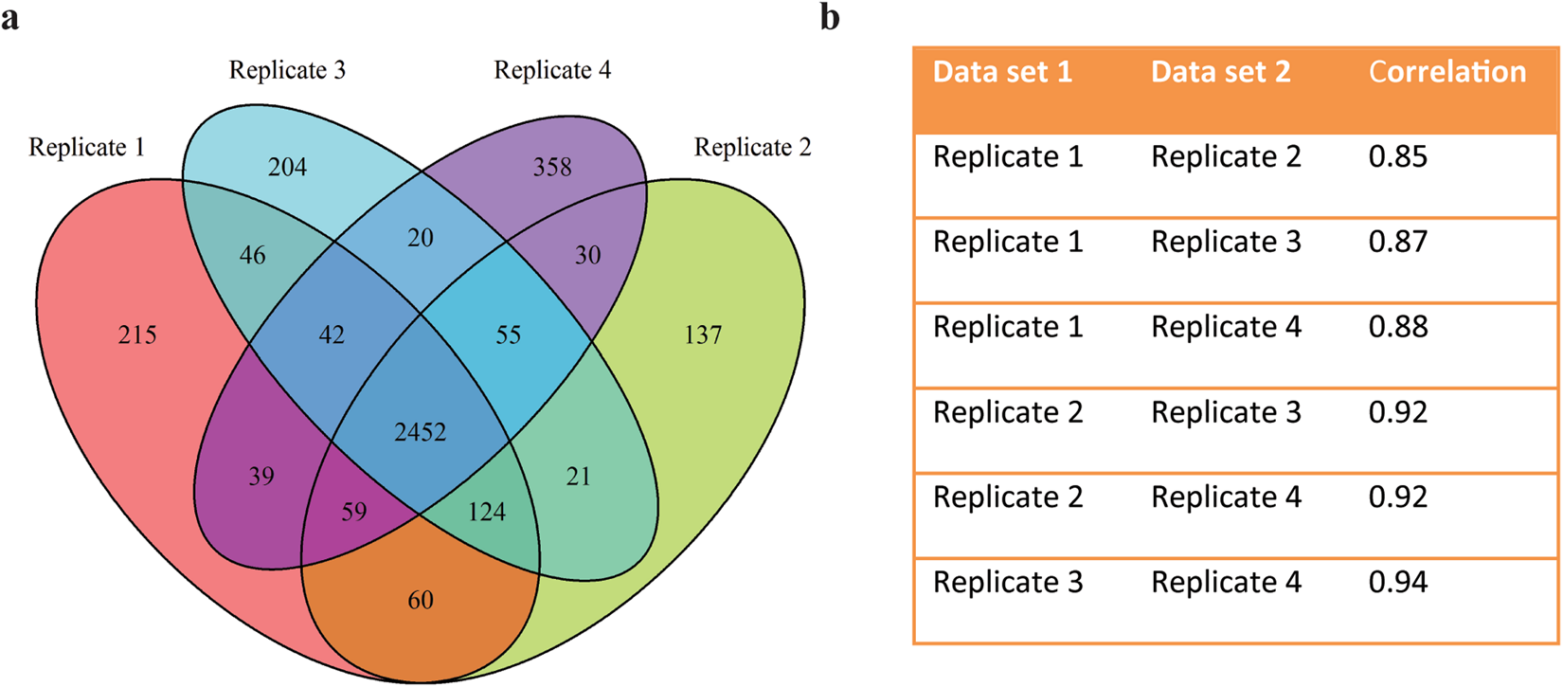
Evaluation of the HiT-Gel method on native protein extracts separated by blue native PAGE. Four biological replicates of native *A. thaliana* protein extracts were separated by BN-PAGE and prepared for LC-MS/MS using the HiT-Gel method. **(a)** The overlap in quantified proteins between all four replicates is shown as a venn diagram. **(b)** The mean correlation factors for proteins quantified in each of the replicates were calculated and are presented here.

**Figure 4:**
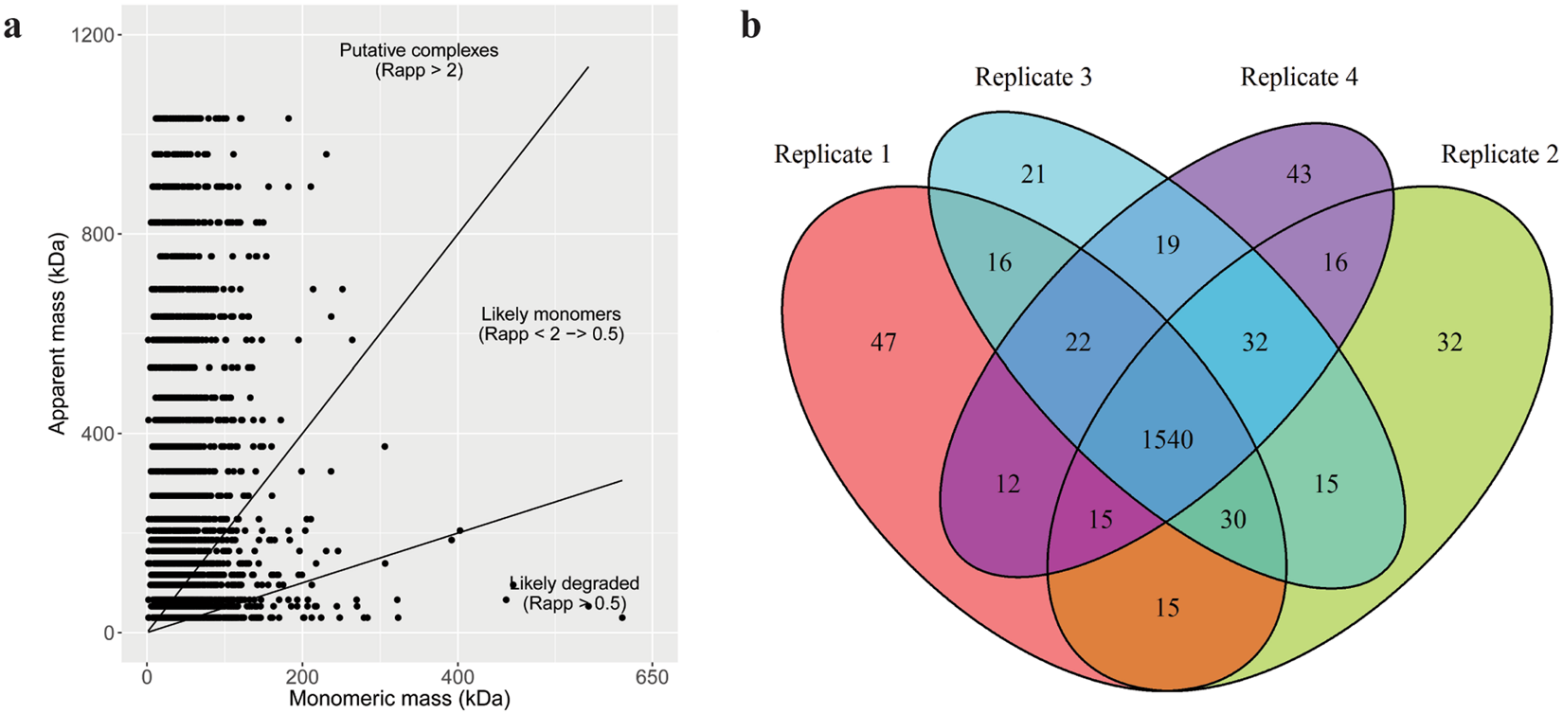
Identification of putative protein complexes using the HiT-Gel method. **(a)** The distribution of the apparent mass of the quantified proteins is shown as a scatterplot. Circles are representative of the apparent mass of the proteins (y-axis) as identified experimentally, in comparison to their TAIR10 annotated monomeric masses (x-axis). Rapp values indicate the oligomerization state of the proteins. Rapp values >2 indicate putative complexes; Rapp values of between 0,5 and 2 describe proteins that likely exist as monomers; Rapp values of <0,5 likely represent degraded proteins. **(b)** The overlap between proteins found to exist as a complex is shown between the four biological replicates as a venn diagram.

Using the HiT-Gel method, four 96-well plates can be handled in parallel. Hence, the newly established method allows for the first time the simultaneous preparation of up to 384 sample fractions by in-gel digestion. Such high-throughput of samples could be of interest for large-scale proteomics approaches with high sample replication or detailed fractionation. Analysis of protein complexes by native PAGE and mass spectrometry is an example where each biological or technical replicate will be cut into 20 to 30 sample fractions following native PAGE. To test if HiT-Gel can facilitate large-scale studies of protein complexes, soluble native proteins were extracted from Arabidopsis rosettes in 4 biological replicates. Following separation of the protein extracts by native PAGE, coomassie staining was performed and each of the replicates was cut into 24 fractions representing a size range from 30 to 1032 kDa (Supplementary Figure S5). Hence, in total 96 gel fractions were created and subjected to the HiT-Gel method and analysed by HPLC-MS/MS. In total 3861 proteins were detected and 2452 were identified in all 4 biological replicates (Fig. 3a and Supplementary Dataset 3). A correlation analysis was performed to compare the 4 replicates. The correlation was very high ranging from 0.85 to 0.94 in the pairwise comparisons (Fig. 3b). To obtain a profile of protein abundance across the full size range, the mean abundance was calculated for each protein in each of the 24 fractions. Using the statistical software R, peaks in abundance were detected for each protein. The average number of detected peaks per protein was 1.19. To determine how many proteins were detected in protein complexes, a size cut-off was applied. Abundance peaks in size fractions larger than 2-fold the monomeric molecular weight of the protein were considered indicative of protein complex formation. Using this cut-off, 2581 proteins (67% of proteins quantified in all replicates) were identified in a complex (Fig. 4a and b). Moreover, 1402 proteins had peak abundance in the size range between 0.5 and 2-fold the molecular weight and were considered monomeric (Fig. 4a). Peaks in size fractions smaller than 0.5-fold the monomeric weight, were considered degradation products. This was observed for 11 proteins.

The proteasome is regularly used as a quality control for datasets analysing native protein complexes^14–16^. This complex, which comprises of a catalytic 20S core particle (CP) and a 19S regulatory particle (RP), is responsible for the degradation of proteins in the cytosol and can be found in all eukaryotic cells^17^. Using the KEGG^18^ database annotation of the Arabidopsis proteasome we created a heatmap of all proteasome subunits identified in the present study (Supplementary Figure S6). The heatmap shows that the regulatory and the core particle of the proteasome are clearly separated by native PAGE. Moreover, the 2 particles are represented with almost complete coverage. Out of 23 proteins annotated in the core particle, 21 were detected. For the regulatory particle 23 proteins out of 32 annotated in KEGG were identified. To our knowledge such a high coverage of the Arabidopsis proteome has not been reported in a proteome wide study of protein complexes. In summary, the HiT-Gel method facilitates the analysis of protein complexes by native PAGE with low sample to sample variation and high coverage. The advantage of this method over the commonly used size exclusion chromatography is, that biological replicates can be analysed in parallel on one gel. Consequently, native PAGE size fractionation in conjunction with HiT-Gel sample preparation and HPLC-MS/MS analysis allows quantitative comparative studies of changes in protein complex abundance and/or composition between different treatments or genotypes.

## Conclusion

We have modified the conventional in-gel digestion approach to facilitate high-throughput sample preparation. Using the HiT-Gel method, up to four 96-well plates, corresponding to 384 sample fractions, can be processed in parallel with ease. Assuming that 48 reaction tubes could be handled in parallel, 384 sample fractions would require 8 independent runs through the conventional method. The reduced handling of samples results in a lower sample-to-sample variation and contamination level compared to the conventional method. Our data shows that, although previously considered a prerequisite for the conventional in-gel digestion approach, the subdivision of gel fractions into 1 mm × 1 mm cubes is not required for efficient peptide recovery. In fact, the HiT-Gel method outperforms the conventional method better in terms of peptide recovery from the gel matrix. Finally, we have also demonstrated that the HiT-Gel method can be applied to the study of protein complexes on a scale that would previously have been considered as excessively labour intensive.

## Methods

### Plant growth conditions and sample preparation procedures

Wild-type *A. thaliana* Col-0 plants were cultivated on soil for 3 weeks in a controlled environment room under a neutral-day photoperiod (12 h light/12 h dark, 130 µmol photons m^−2^ s^−1^, 60% ambient humidity and 20 °C). Biological replicates were prepared by combining whole rosettes of 5 plants. Plant tissue was harvested into liquid nitrogen before being ground to a fine powder. Proteins were extracted under denaturing or native conditions as specified in the experimental workflow. Under denaturing conditions, a SDS buffer (4% [w/v] sodium dodecyl sulfate (SDS); 40 mM Tris-Cl pH 7.5; 5% [v/v] glycerol; 30 µl.ml^−1^ Protease Inhibitor Cocktail (Roche)), was added to the sample in a 1:1, 5 weight to volume ratio and the proteins extracted at room temperature. Under native conditions a native extraction buffer (25 mM Tris-Cl, pH 7,0; 20% [v/v] glycerol; 30 µl.ml^−1^ Protease Inhibitor Cocktail (Roche)) was added in the same ratio as mentioned before. Estimation of protein concentration was performed with the aid of a BCA assay kit (Thermo Scientific) according to the instructions of the manufacturer.

### Protein separation by SDS-PAGE

Approximately 100 µg of SDS-extracted *A. thaliana* proteins or 50 ng of BSA was used for each of the technical replicates. Protein samples were separated under denaturing conditions on a Mini-Protean handcast 12% Tris-glycine gels with a 5% stacking gel (1 mm spacer width) using the BioRad Mini-PROTEAN® system.

### Protein separation by blue native PAGE

Approximately 300 µg of *A.thaliana* native protein extract was used for each of the technical replicates. Protein sample separation was performed, under native conditions, on a large format handcast blue native Tris-glycine gel (16 × 20 cm, gradient ranging from 3,5% to 9%) with the BioRad PROTEAN® II xi cell.

### Gel staining and in-gel digestion

After protein separation on by PAGE, staining was performed at room temperature for one hour using a Coomassie Blue solution (20% [v/v] methanol; 10% [v/v] acetic acid; 0,1% [w/v Coomassie Brilliant Blue R]). Destaining of the gels was subsequently performed at room temperature for 2 hours (or 6 hours for large gels) using a destaining solution (50% [v/v] methanol; 10% [v/v]). On mini gels, each sample was fractionated into 8 gel pieces, while samples on large format gels were cut into 24 fractions. The conventional method was performed as previously reported^1,2^. The HiT-Gel method is described in detail in the Supplementary Methods. Following the in-gel digestion procedures, the peptides were desalted on Finisterre C18 SPE columns (Teknokroma) by reverse-phase chromatography. Peptides were dried using a vacuum centrifuge before storage at −80 °C.

### Liquid chromatography and mass spectrometry analysis

Peptides were resuspended in 30 μl of resuspension buffer (5% v/v acetonitrile, 2% v/v trifluoroacetic acid). Measurements were performed on a Q Exactive HF coupled to an Easy nLC1000 HPLC (Thermo Scientific). 8 μl of the samples were loaded onto an Acclaim PepMap RSLC reversed-phase column (75 μm inner diameter, 15 cm length, 2 µm bead size (Thermo Scientific)) at a flow rate of 0.4 μl min^−1^ in a buffer consisting of 3% (v/v) acetonitrile, 0.5% (v/v) acetic acid. Peptide elution was performed by increasing the acetonitrile gradient from 3% to 30% [v/v] over 110 min, from 30% to 40% for the next 10 min and from 40% to 80% for the last 1 min. The column was then washed with 80% [v/v] acetonitrile for 4 min, at a flow rate of 0.3 μl/min. Peptide ions were detected in a full MS1 scan for mass to charge ratios between 300 and 1600 m/z at a resolution of 70 000. Data dependent tandem mass spectrometry scans were performed at a resolution of 17 500 for the 15 most abundant ions (AGC target 2e5, isolation width mass-to-charge ratio, 2 m/z, relative collision energy, 30%). Peptides for which MS/MS spectra had been recorded were excluded from further MS/MS scans for 20 seconds.

### Peak area based protein quantification and statistical analysis for the method comparison

Quantitative analysis of MS/MS measurements was performed with the Progenesis QI software (Nonlinear Dynamics). Proteins were identified from the raw spectra using Mascot (Matrix Science). Mascot search parameters were: *A. thaliana* TAIR10 protein annotation^19^, requirement for tryptic ends, one missed cleavage allowed, fixed modification: carbamidomethylation (cysteine), variable modification: oxidation (methionine), peptide mass tolerance = ±10 ppm, MS/MS tolerance = ±0.6 Da, allowed peptide charges of +2 and +3. Only unique peptides were considered and no protein grouping was selected. A decoy database search was used to limit false discovery rates to 1% on the peptide level. Peptide identifications below rank one or with a Mascot ion score below 25 were excluded. Mascot results were imported into Progenesis QI, quantitative peak area information extracted and the results exported to R statistical software for data plotting and statistical analysis.

### Peak area based protein quantification and statistical analysis for the study of protein complexes

Protein identification and quantitation based on the raw spectral data was performed using MaxQuant^20^. The Andromeda search engine in combination with the TAIR 10 protein sequence database was used for peptide identification. The following parameters were selected for the search: peptide mass tolerance = ±10 ppm, MS/MS tolerance = ±0.8 Da, a maximum of two missed cleavages allowed, a threshold of 0.01 for the validation of peptides using a decoy database, carbamidomethylation of cysteine was set as a fixed modification and the oxidation of methionine was set as a variable modification. The “label-free quantification” and “match between runs” options were also highlighted for the search. Only peptides with a length of 6 amino acids or more were considered valid. Protein quantification was performed when at least one unique and one razor peptide was present. Known contaminants, such as keratin, were excluded from the analysis. The processed data was exported to R statistical software for visualisation and statistical analysis.

### Data analysis in R statistical software

Data was analysed in R studio (R-3.3.3, 64 bit). Analyses were performed at the protein level except where clearly stated the peptide information was used. Proteins that appeared in 2 out of the 3 replicates were used for the comparison of the protocols. The covariance was plotted based on the kernel density estimates and excluded contaminants. The overlapping proteins, detected across all of the replicates, were used for the study of protein complexes. Each of the protein’s profiles were normalized to their maximum abundance before the Pearson correlation coefficient was calculated, in a pairwise manner, between the replicates using the “cor” function in R. The mean value was used as an indicator of the absolute correlation between replicates. Venn diagrams were generated using the “venn.diagram” function from the VennDiagram package in R. To estimate the reproducibility between the replicates, the coefficient of variation was calculated for all overlapping proteins in the individual replicates. This was performed using the “density” function in R. Histograms with normal curve were generated using the “hist” function in R. Where required the “ggplot2” and “easyGgplot2” packages were used for data visualisation.

### Data availability

The proteomics datasets produced in this study are available in the PRIDE database using the following accessions: SDS PAGE protocol comparison: PXD009510 Native PAGE analysis: PXD009485.

## Electronic supplementary material

Supplementary Figures and Tables

Supplementary Methods

Supplementary Dataset 1

Supplementary Dataset 2

Supplementary Dataset 3
