# Supplementary material for: Hit-Gel: Streamlining in-gel protein digestion for high-throughput proteomics experiments
Source: Sci Rep. 2018 Jun 5;8:8582. doi: 10.1038/s41598-018-26639-3 (PMC5988721; doi:10.1038/s41598-018-26639-3)
Supplement: Supplementary file 1 — Supplementary Figures and Tables [file 41598_2018_26639_MOESM1_ESM.pdf]

Hit-Gel: Streamlining in-gel protein digestion for high-throughput proteomics experiments.

Corné Swart[1¶], Silvia Martínez-Jaime[1¶], Michal Gorka[1], Kerstin Zander[1] and Alexander Graf[1*]

[1]Max Planck Institute of Molecular Plant Physiology, 14476 Potsdam-Golm, Germany

[*]Corresponding author:

Email: graf@mpimp-golm.mpg.de (AG)

[¶]These authors contributed equally to this work.

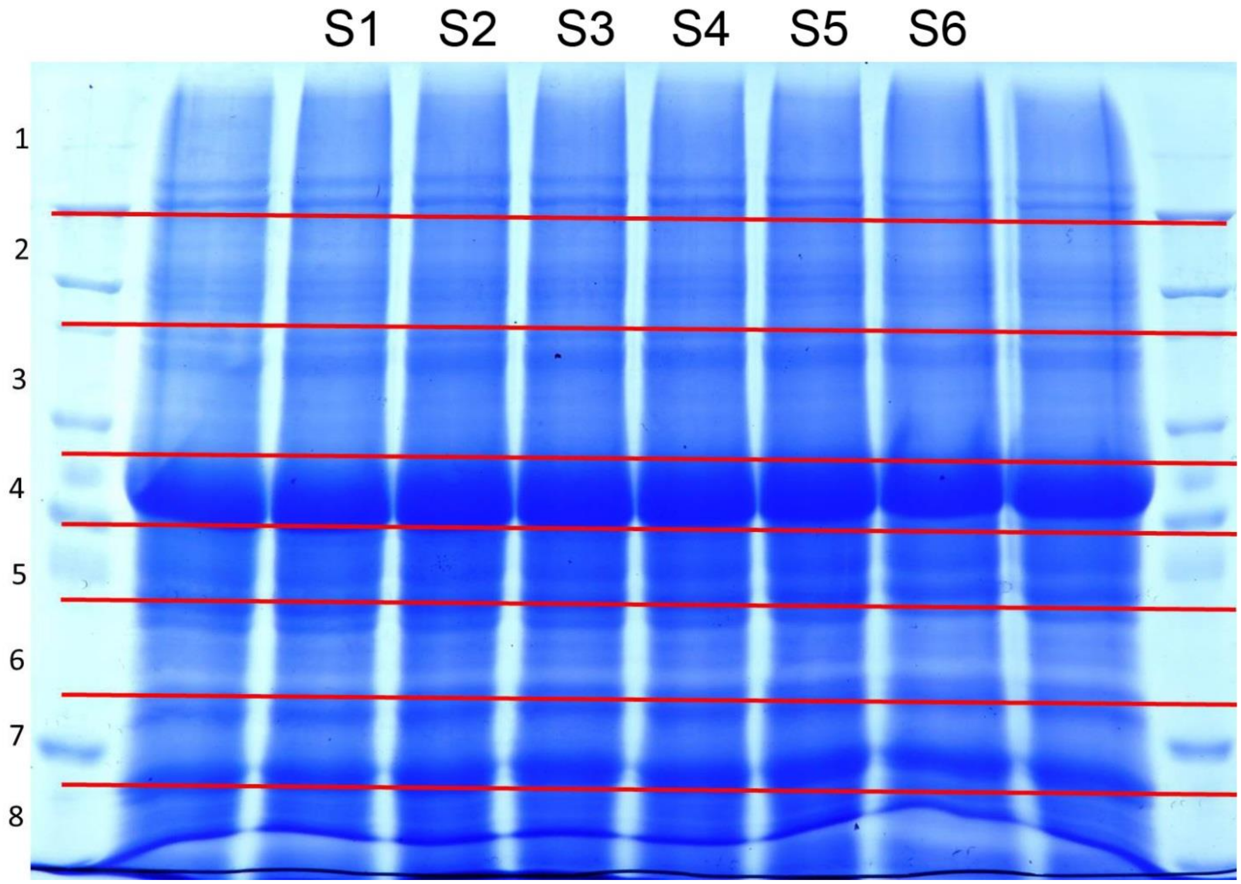

Supplementary Figure S1. SDS PAGE gel of complex protein extracts with cutting guide. Photograph depicting the 12% SDS-PAGE gel used to separate the complex protein extract. Lanes prepared for in-gel digestions are labelled S1 to S6. Lanes S1, S3 and S5 were used for the conventional in-gel digestion. Lanes S2, S4 and S6 were prepared for MS by HiT-Gel. The fractionation guidelines (red horizontal lines) to aid the cutting procedure have been added using a graphical editing tool.

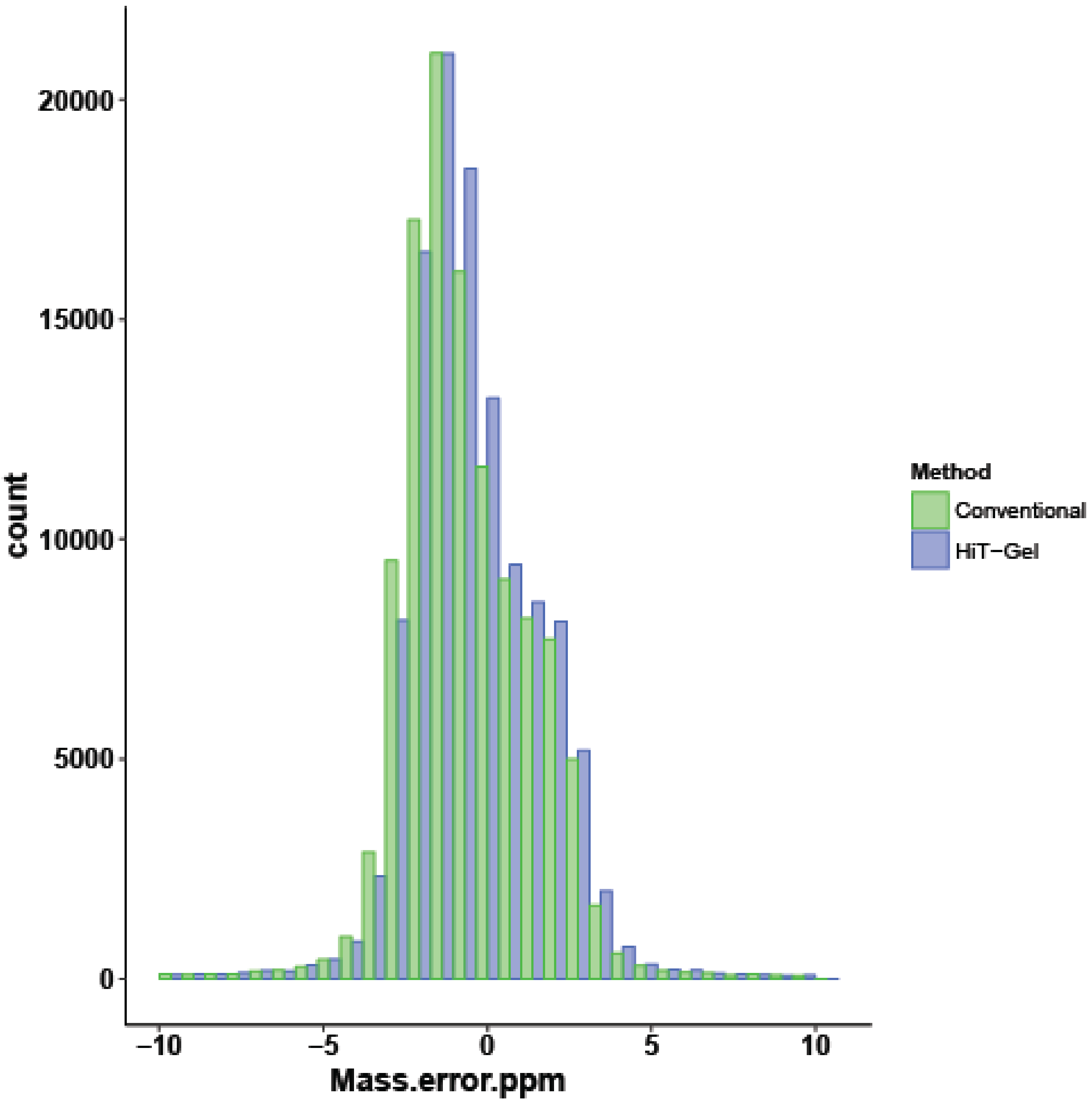

Supplementary Figure S2. Mass error distribution for the HiT-Gel and the conventional method. The mass error distribution, for each of the methods, was exported from Progenesis QI and presented as a bar plot.

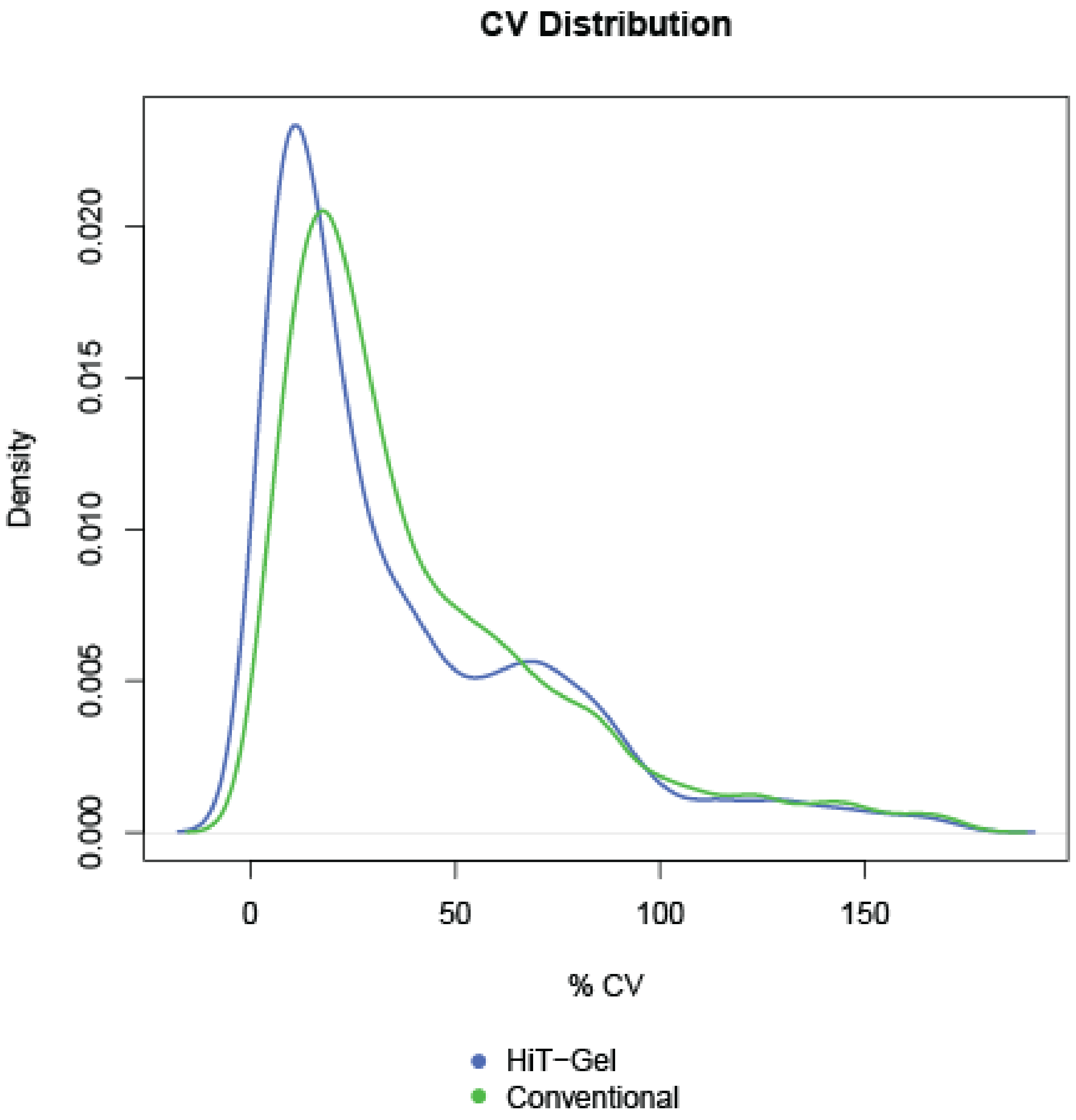

Supplementary Figure S3. Covariance distribution for the HiT-Gel and the conventional method. The covariance was calculated for all proteins in each of the two datasets and a density plot was created to visualize the CV distribution.

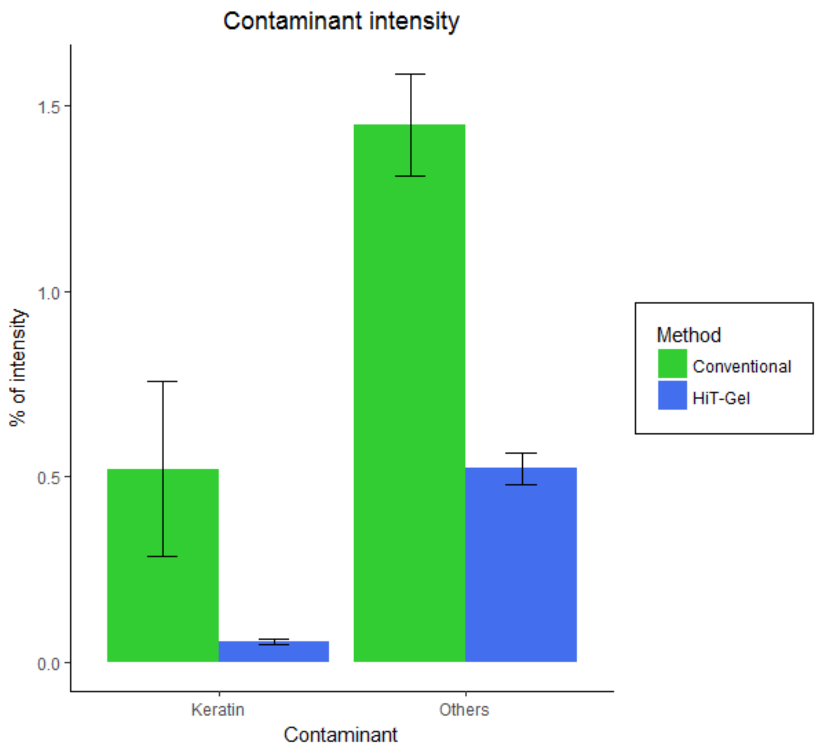

Supplementary Figure S4. Relative signal intensities of contaminants in the complex extracts analysed using the Hit-Gel and conventional methods. The total signal of the contaminants was divided into two groups. The first group represents the signal intensity that originates from peptides that are derived from keratin, while the second group is reserved for all other contaminant peptides. The bar plot shows the signal intensity of contaminants relative to the total ion intensity obtained from the samples prepared with the conventional (green bars) and the HiT-Gel method (blue bars). Error bars represent the standard error, n=3.

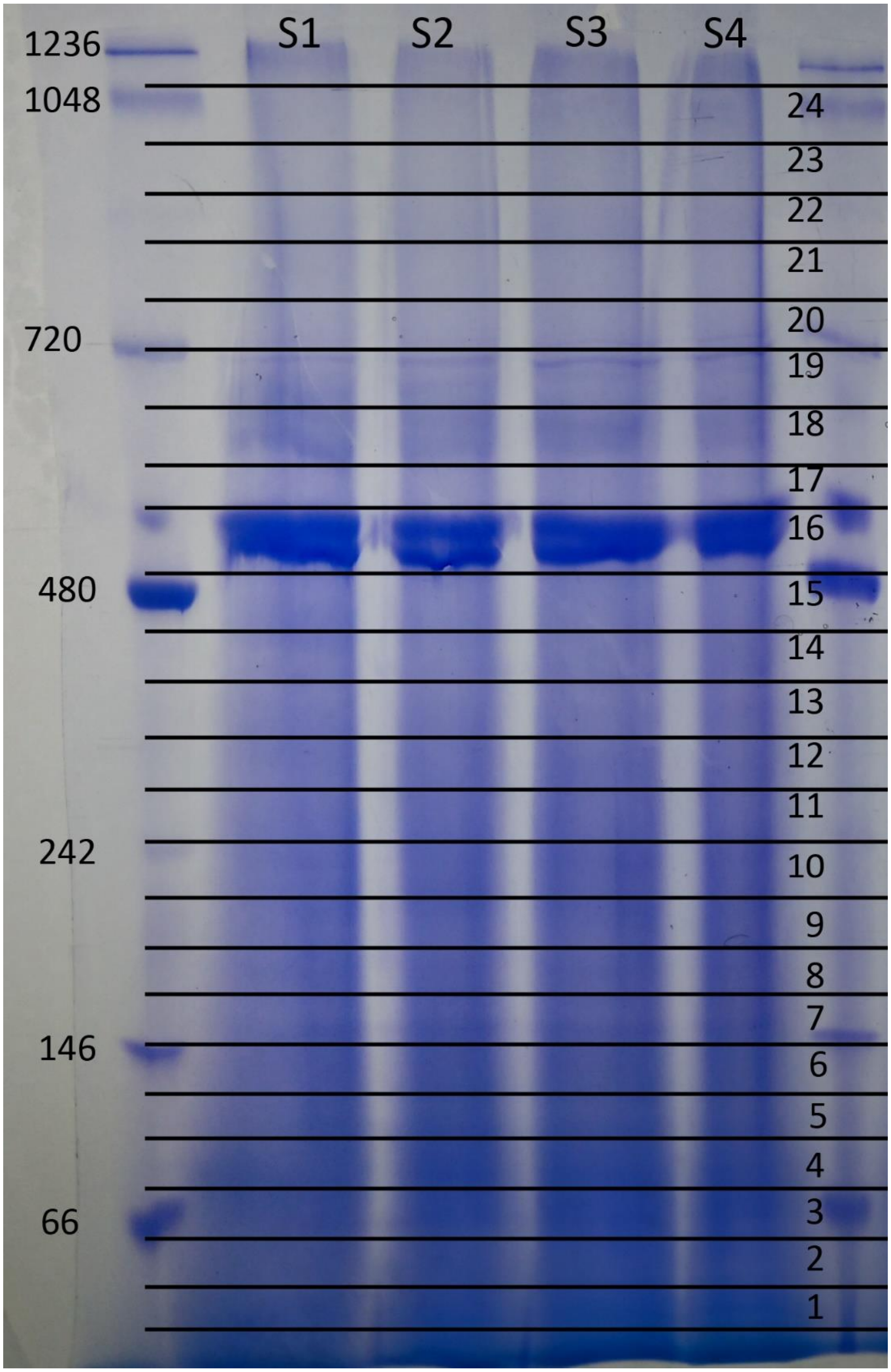

Supplementary Figure S5. Blue native PAGE gel of complex protein extracts with cutting guide. Photograph depicting the 3,5% to 9% gradient blue native Tris-glycine gel used to separate the native protein extracts. Lanes prepared for in-gel digestions are labelled S1 to S4. The fractionation guidelines (black horizontal lines) to aid the cutting procedure have been added using a graphical editing tool.

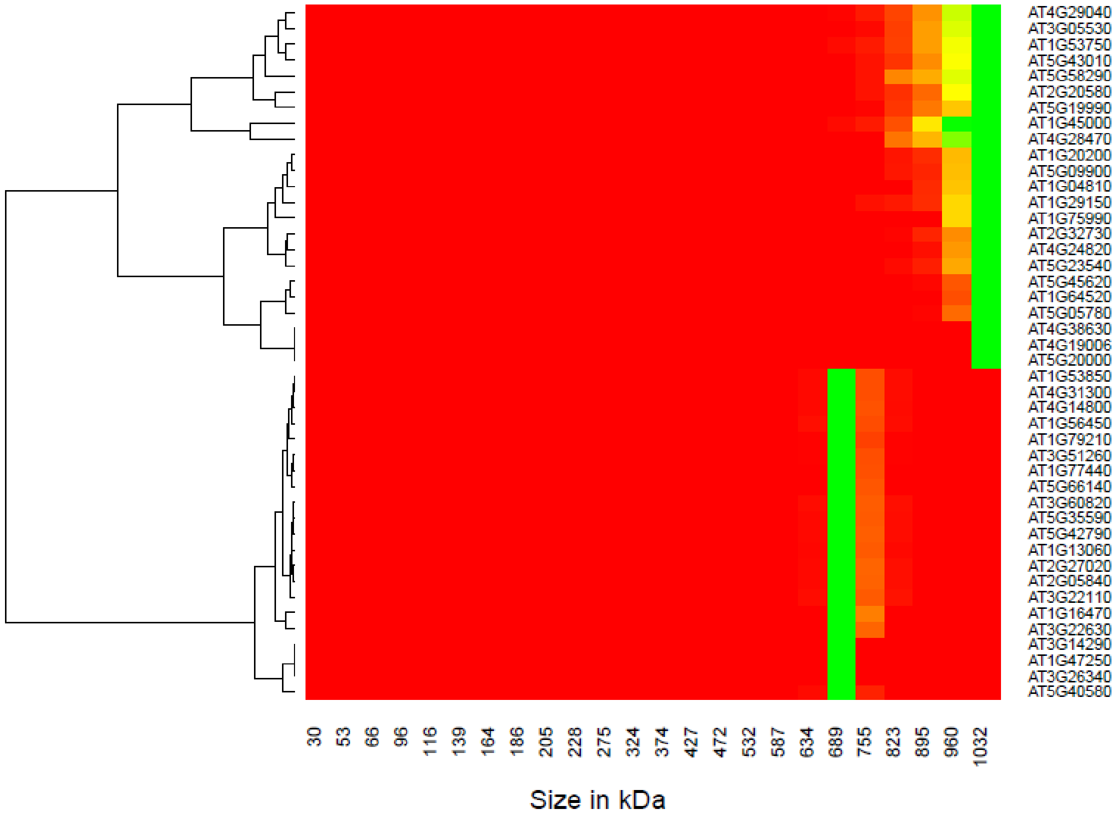

Supplementary Figure S6. Validation of protein complex detection using the proteasome. The proteins from our native-PAGE dataset that belong to the regulatory (upper cluster) and core (lower cluster) subunits of the proteasome were observed to cluster together. For each protein, mean signal intensities of the 4 biological replicates were plotted. The green areas indicate the size range in which the highest abundance of the individual proteins were found.

Supplementary Table S1. Analysis of bivariate correlation within the technical replicates of the conventional in-gel digestion method. The mean correlation factors for proteins quantified in each of the replicates of the conventional in-gel digestion were calculated using the Pearson correlation coefficient approach and are presented here.

Data set 1	Data set 2	Correlation
Replicate 1	Replicate 2	0.99
Replicate 1	Replicate 3	0.98
Replicate 2	Replicate 3	0.99

Supplementary Table S2. Analysis of bivariate correlation within the technical replicates of the HiT-Gel method. The mean correlation factors for proteins quantified in each of the replicates of the HiT-Gel method were calculated using the Pearson correlation coefficient approach and are presented here.

Data set 1	Data set 2	Correlation
Replicate 1	Replicate 2	0.99
Replicate 1	Replicate 3	0.98
Replicate 2	Replicate 3	0.98

Supplementary Table S3. Analysis of bivariate correlation between the technical replicates of the HiT-Gel and the conventional method. The mean correlation factors for proteins quantified in each of the replicates of the HiT-Gel method and the conventional method were calculated using the Pearson correlation coefficient approach and are presented here.

HiT-Gel	Conventional	Correlation
Replicate 1	Replicate 2	0.99
Replicate 1	Replicate 2	0.99
Replicate 1	Replicate 3	0.99
Replicate 2	Replicate 1	0.98
Replicate 2	Replicate 2	0.99
Replicate 2	Replicate 3	0.99
Replicate 3	Replicate 1	0.99
Replicate 3	Replicate 2	0.98
Replicate 3	Replicate 3	0.98

Supplementary Table S4. Comparison of the HiT-Gel and conventional method using a single protein band. Protein fractions containing BSA were excised from the gel and analysed using the HiT-Gel method and the conventional method. The number of unique peptides that were detected for BSA was reported in conjunction with their total signal intensity. Additionally, the signal intensity for the BSA protein is shown.

	HiT-Gel	Conventional
**Number of quantified BSA peptides**	38	33
**Intensity of BSA peptides**	4.4e7	1.76e7
