# Supplementary material for: Hit-Gel: Streamlining in-gel protein digestion for high-throughput proteomics experiments
Source: Sci Rep. 2018 Jun 5;8:8582. doi: 10.1038/s41598-018-26639-3 (PMC5988721; doi:10.1038/s41598-018-26639-3)
Supplement: Supplementary file 2 — Supplementary Methods [file 41598_2018_26639_MOESM2_ESM.docx]

**Hit-Gel: Streamlining in-gel protein digestion for high-throughput proteomics experiments.**

Corné Swart^1¶^, Silvia Martínez-Jaime^1¶^, Michal Gorka^1^, Kerstin Zander^1^ and Alexander Graf^1*^

^1^Max Planck Institute of Molecular Plant Physiology, 14476 Potsdam-Golm, Germany

^*^Corresponding author:

Email: [graf@mpimp-golm.mpg.de](mailto:graf@mpimp-golm.mpg.de) (AG)

^¶^These authors contributed equally to this work.

**Supplementary methods:**

**A comprehensive guide to the HiT-Gel method**

**Required reagents:**

Ammonium Bicarbonate (Sigma-Aldrich)

Dithiothreitol (DTT) (Duchefa Biochemie)

Iodoacetamide (IAM) (Sigma-Aldrich)

Distilled water

Sequencing Grade Modified Trypsin (Promega)

Methanol (LiChrosolv® Merck Millipore)

Trifluoroacetic acid (TFA) (Sigma-Aldrich)

Acetonitrile (LiChrosolv® Merck Millipore)

**Equipment used:**

Light-box (Type 960/1, Deitenbach)

18mm Standard Snap-Off knife blades (STANLEY Tools)

Pipet-Lite Pipette Multi L8-1200XLS+ (Rainin - Solutions by Mettler Toledo)

LTS 1000 µl tips (Rainin - Solutions by Mettler Toledo)

2 ml PlateOne® 96 Deepwell plate, Square with V-shape Bottoms (STARLAB)

2 ml reaction tubes (Eppendorf® Safe-Lock microcentrifuge tubes, Sigma-Aldrich)

Vacuum centrifuge with 96 Deepwell plate compatible swingout rotor (Savant™ SPD131DDA SpeedVac™ Concentrator with Thermo RVT400 Refrigerated Vapor Trap, Thermo Fisher Scientific)

Incubator or oven set to 37°C (Binder BD 53)

Water bath set to 50°C (GFL Water Bath Type 1003)

Orbital shaker (GFL 3017)

Vacuum pump with dessicator (Diaphragm Vacuum Pump Laboport® N 810.3 FT.18 KNF with Nalgene™ Transparent Polycarbonate Classic Design Dessicator)

**Reagent setup:**

- 50 mM ammonium bicarbonate in water. **Critical:** Prepare fresh shortly before use.
- **DTT solution**: 10 mM DTT in 5mM ammonium bicarbonate solution. **Critical:** prepare fresh shortly before use.
- **IAM Solution**: 50 mM IAM in 50mM ammonium bicarbonate. **Critical:** Prepare fresh shortly before use.
- **Destaining Buffer:** 50 mM ammonium bicarbonate in 50% (v/v) methanol. **Critical**: Prepare fresh before use.
- **Trypsin solution:** Approximately 1 µg of trypsin is required for every 30 µg of protein. Prepare the appropriate amount of trypsin in a 50mM ammonium bicarbonate solution.

**Procedure**

**Overview**

**Step 1-6 Fractionation of intact gels**

**Step 7- 9 Destaining of gel pieces**

**Step 10-30 Protein tryptic digest**

**Step 31-35 Collection of peptides (Divided into options A, B and C)**

**Fractionation of intact gels**

1. Visualize the intact gel and determine the number and size of fractions based on the amount of protein samples. Take a picture of the gel and use a graphical editing tool to plan the fractionation as in Fig. S1. This improves reproducibility and decreases technical variation.
2. Pipette 900 µl of distilled water into the wells of a 96-well multiwell plate.

**!Critical step**. This prevents gel pieces from drying out.

1. Divide intact gels into 8 horizontal strips of approximately equal size. Use a Snap-Off Knife blade (or similar long blade) to slice each strip with a single slicing action to reduce technical variation.
2. Use a sharp blade and further divide the horizontal strips vertically using the boundaries created by the lanes of each protein sample.
3. Transfer each of the rectangular gel fractions to a new well in the 96-well plate prepared in the first step. Accurately track where each fraction is now represented in the 96-well plate.
4. Use a pipette and remove the distilled water from each well.

!**Critical step.** Take care not to damage, transfer or lose gel pieces during the pipetting steps.

**PAUSE POINT** – At this stage the multiwell plates can be stored at -20°C until further processing.

**Destaining of gel pieces**

1. Pipette 900 µl of **Destaining buffer** into each well containing a gel fragment.
2. Incubate the samples on an orbital shaker (180 RPM) for at least 20 minutes at 37°C.
3. Remove the **Destaining buffer** with pipette and repeat step 7 and 8 at least two more times. When the **Destaining buffer** no longer turns blue, the gel pieces are sufficiently destained. Excessively stained gel pieces might require additional destaining. See troubleshooting.

**PAUSE POINT** – At this stage the multiwell plates can be stored at -20° C until further processing.

**Protein tryptic digest**

1. Using a multichannel pipette add 900 µl of 50% (v/v) acetonitrile to each sample well and incubate for 15 minutes at room temperature.
2. Remove the acetonitrile with the aid of a multichannel pipette.
3. Repeat steps 10 and 11.

**!Critical step.** Gel pieces that have been efficiently dehydrated will shrink and have a whitish appearance. See **Troubleshooting** when the dehydration step does not appear effective.

1. Place the 96-well multiwell plate in a dessicator with an attached vacuum pump. Switch the vacuum on for at least 40 minutes.
2. Preheat the **DTT solution** to 50°C in a water bath.
3. Release the vacuum and remove the 96-well multiwell plate from the dessicator.

**!Critical step.** Visually inspect the wells for traces of liquid that might interfere with the downstream tryptic digest. See **Troubleshooting**.

1. Add 600 µl of preheated **DTT solution** to the sample wells using a multichannel pipette and incubate at 50°C in a water bath for 45 minutes.
2. Remove the **DTT solution** from the sample wells with a multichannel pipette.
3. Add 600 µl of IAM solution to each sample well with the aid of a multichannel pipette and incubate for 1 hour and 45 minutes at room temperature in the dark.
4. Remove the **IAM solution** from the sample wells using a multichannel pipette.
5. Use a multichannel pipette and add 900 µl of 50% (v/v) acetonitrile to each sample well. Incubate for 15 minutes at room temperature.
6. Remove the acetonitrile with the aid of a multichannel pipette.
7. Repeat steps 20 and 21.

**!Critical step**. Gel pieces that have been efficiently dehydrated will shrink and have a whitish appearance. See **Troubleshooting** when the dehydration step does not appear effective.

1. Place the 96-well multiwell plate in a dessicator with an attached vacuum pump. Switch the vacuum on for at least 40 minutes.
2. Release the vacuum and remove the 96-well multiwell plate from the dessicator.

**!Critical step**. Visually inspect the wells for traces of liquid that might interfere with the downstream tryptic digest. See **Troubleshooting**.

1. Add 300 µl of cold **Trypsin solution** to each sample well using a multichannel pipette.
2. Centrifuge the 96-well multiwell plate at 1000 x g for 1 minute at 4°C.
3. Incubate the 96-well multiwell plate at 4°C for 30 minutes.
4. Add 200 µl of 50mM of ammonium bicarbonate to the **Trypsin solution** in each sample well with the aid of a multichannel pipette and incubate overnight at 37°C.

**!Critical step.** The gel pieces have to be fully immersed in the solution. Small gel pieces might be fully immersed following step 27. In this case step 28 can be omitted. If gel pieces are not be fully immersed after step 28, more 50mM of ammonium bicarbonate should be added to the respective well. The volumes in step 29 and 30 should be adjusted accordingly.

1. Use a multichannel pipette and add 30 µl of 10% (v/v) trifluoroacetic acid to each sample well.
2. Add a further 500 µl of neat acetonitrile to each sample well with the aid of a multichannel pipette and incubate for 20 minutes at room temperature.

**At this stage the user can select any of the options, A to C, based on the availability of laboratory equipment.**

**Option A**

1. Use a multichannel pipette and transfer the supernatant in each sample well to the corresponding well of a new 96-well multiwell plate.
2. Pipette 300 µl of 2% (v/v) trifluoroacetic acid on top of the now exposed gel pieces in the original 96-well multiwell plate with the aid of a multichannel pipette. Incubate for 20 minutes at room temperature.
3. Add a further 400 µl of neat acetonitrile to each sample well containing a gel piece using a multichannel pipette. Incubate for 30 minutes at room temperature.
4. Transfer the supernatant from each well containing a gel piece using a multichannel pipette and combine it with the supernatant in the new 96-well multiwell plate introduced in step 31.
5. Load the 96-well multiwell plate containing the supernatant into a vacuum centrifuge with a swing out rotor and dry the samples at 55°C for approximately 4 hours or until no liquid remains.

**PAUSE POINT** – At this stage the multiwell plates can be stored at -20°C for a few weeks or at -80 °C until further processing.

**Option B**

1. Use a multichannel pipette with an adjustable spacer and transfer the supernatant from each sample well to labeled 2 ml reaction tubes that are arranged in a layout corresponding to that of the 96-well plate.

**!Critical step.** The spacer is first adjusted for collecting the supernatant from the wells of the plate. After the supernatant has been pulled up into the tips attached to the multichannel pipette, the spacer width is adjusted to facilitate the delivery of the supernatant into the reaction tubes.

1. Pipette 300 µl of 2% (v/v) trifluoroacetic acid on top of the now exposed gel pieces in the 96-well multiwell plate with the aid of a multichannel pipette. Incubate for 20 minutes at room temperature.
2. Add a further 400 µl of neat acetonitrile to each sample well containing a gel piece using a multichannel pipette. Incubate for 30 minutes at room temperature.
3. Transfer the supernatant from each well containing a gel piece using a multichannel pipette with an adjustable spacer and combine it with the supernatant in the corresponding 2 ml reaction tube.
4. Load the reaction tubes into a vacuum centrifuge and dry the samples at 55°C for approximately 4 hours or until no liquid remains.

**PAUSE POINT** – At this stage the reaction tubes can be stored at -20°C for a few weeks or at -80 °C until further processing.

**Option C**

1. Use a standard pipette and transfer the supernatant from each sample well to labeled 2 ml reaction tubes that are arranged in a layout corresponding to that of the 96-well plate.
2. Pipette 300 µl of 2% (v/v) trifluoroacetic acid on top of the now exposed gel pieces in the 96-well multiwell plate with the aid of a standard pipette. Incubate for 20 minutes at room temperature.
3. Add a further 400 µl of neat acetonitrile to each sample well containing a gel piece using a standard pipette. Incubate for 30 minutes at room temperature.
4. Transfer the supernatant from each well containing a gel piece using a standard pipette and combine it with the supernatant in the corresponding 2 ml reaction tube.
5. Load the reaction tubes into a vacuum centrifuge and dry the samples at 55°C for approximately 4 hours or until no liquid remains.

**PAUSE POINT** – At this stage the reaction tubes can be stored at -20°C for a few weeks or at -80 °C until further processing.

**Proceed with peptide cleanup procedure of choice**

**Troubleshooting:**

**Step 9: Inefficient destaining.** This can be solved by not only increasing the incubation time, but also by replacing the buffer more frequently.

**Steps 12 and 22**: **Ineffective dehydration of gel pieces with acetonitrile.** The acetonitrile concentration can be incrementally increased up to 80%.

**Steps 15 and 24**: **Incomplete evaporation of acetonitrile.** The samples can be returned to the vacuum dessicator until all the liquid has evaporated.
